# Nitazoxanide inhibits the replication of Japanese encephalitis virus in cultured cells and in a mouse model

**DOI:** 10.1186/1743-422X-11-10

**Published:** 2014-01-23

**Authors:** Zixue Shi, Jianchao Wei, Xufang Deng, Shuqing Li, Yafeng Qiu, Donghua Shao, Beibei Li, Keyu Zhang, Feiqun Xue, Xiaodu Wang, Zhiyong Ma

**Affiliations:** 1 Shanghai Veterinary Research Institute, Chinese Academy of Agricultural Science, No. 518, Ziyue Road, Shanghai 200241, PR China; 2 Shanghai Entry-Exit Inspection and Quarantine Bureau, No. 1208, Minsheng Road, Shanghai 200135, PR China; 3 Forestry and Biotechnology School, Zhejiang Agriculture and Forestry University, Lin’an, Hangzhou, China

**Keywords:** Japanese encephalitis virus, Nitazoxanide (NTZ), Antiviral

## Abstract

**Background:**

Japanese encephalitis virus (JEV) has a significant impact on public health. An estimated three billion people in 'at-risk’ regions remain unvaccinated and the number of unvaccinated individuals in certain Asian countries is increasing. Consequently, there is an urgent need for the development of novel therapeutic agents against Japanese encephalitis. Nitazoxanide (NTZ) is a thiazolide anti-infective licensed for the treatment of parasitic gastroenteritis. Recently, NTZ has been demonstrated to have antiviral properties. In this study, the anti-JEV activity of NTZ was evaluated in cultured cells and in a mouse model.

**Methods:**

JEV-infected cells were treated with NTZ at different concentrations. The replication of JEV in the mock- and NTZ-treated cells was examined by virus titration. NTZ was administered at different time points of JEV infection to determine the stage at which NTZ affected JEV replication. Mice were infected with a lethal dose of JEV and intragastrically administered with NTZ from 1 day post-infection. The protective effect of NTZ on the JEV-infected mice was evaluated.

**Findings:**

NTZ significantly inhibited the replication of JEV in cultured cells in a dose dependent manner with 50% effective concentration value of 0.12 ± 0.04 μg/ml, a non-toxic concentration in cultured cells (50% cytotoxic concentration = 18.59 ± 0.31 μg/ml). The chemotherapeutic index calculated was 154.92. The viral yields of the NTZ-treated cells were significantly reduced at 12, 24, 36 and 48 h post-infection compared with the mock-treated cells. NTZ was found to exert its anti-JEV effect at the early-mid stage of viral infection. The anti-JEV effect of NTZ was also demonstrated *in vivo*, where 90% of mice that were treated by daily intragastric administration of 100 mg/kg/day of NTZ were protected from a lethal challenge dose of JEV.

**Conclusions:**

Both *in vitro* and *in vivo* data indicated that NTZ has anti-JEV activity, suggesting the potential application of NTZ in the treatment of Japanese encephalitis.

## Background

**Figure 1:**
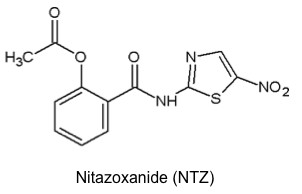
Structure of nitazoxanide (NTZ).

Nitazoxanide (2-acetyloxy-N-(5-nitro-2-thiazolyl) benzamide) (NTZ) (Figure 1) is a thiazolide anti-infective, originally licensed in the United States (Alinia; Romark Laboratories, Tampa, FL, USA), for the treatment of parasitic enteritis caused by *Cryptosporidium parvum* and *Giardia lamblia* in children and adults [1-3]. The antiviral properties of NTZ were discovered during the treatment of cryptosporidiosis in patients with acquired immune deficiency syndrome [4]. Recently, clinical trials have proven the antiviral effectiveness of NTZ in treating rotavirus gastroenteritis in young children, and rotavirus ancd norovirus gastroenteritis in adults [5,6]. In addition, NTZ has been demonstrated to have antiviral properties against hepatitis B virus (HBV), hepatitis C virus (HCV) and human-, avian- and canine-lineage influenza virus [7-10], suggesting that NTZ is a new class of broad-spectrum antiviral drug [11]. In the United States, NTZ is undergoing phase II clinical trials as a combinatorial drug in the treatment of chronic hepatitis C [12].

The mechanism of activity of NTZ against non-viral anaerobic microorganisms is attributed to its interference with pyruvate:ferredoxin oxidoreductase (PFOR) enzyme-dependent electron transfer reactions, which are essential for anaerobic energy metabolism [13]. By contrast, the mechanisms that underlie the antiviral activity of NTZ are not well understood. NTZ has been described to induce PKR (double-stranded-RNA-activated protein kinase) phosphorylation, which leads to the elevation of phosphorylated eIF2α eukaryotic translation initiation factor 2 alpha, an antiviral intracellular protein, in HCV-infected cells [14]. While in influenza virus-infected cells, NTZ prevents the maturation of viral hemagglutinin (HA) protein possibly by blocking HA trafficking between the endoplasmic reticulum and the Golgi complex [9].

Japanese encephalitis (JE), previously known as Japanese B encephalitis, is caused by Japanese encephalitis virus (JEV), an enveloped arbovirus of the *Flavivirus* genus in the family *Flaviviridae*. JE is most prevalent in Southeast Asia and the Far East, and domestic pigs and wild birds are the natural reservoirs of JEV. Transmission of JEV to humans, which occurs mainly via the bite of infected mosquitoes, may cause severe symptoms typically including fever, headache and other incapacitating manifestations [15]. Although two types of JEV vaccines have long been used, JE still causes 10,000-15,000 encephalitic deaths throughout the world annually [16]. As an arthropod-borne infectious disease, JE has recently spread its geographic footprint into previously non-endemic areas [17]. An estimated three billion people in 'at-risk’ regions remain unvaccinated and the number of unvaccinated individuals in certain Asian countries is increasing because of population growth, intensified rice farming, pig rearing, climate change and the lack of vaccination programs and surveillance [17]. Consequently, there is an urgent need for the development of novel therapeutic agents against JE. Although attempts to develop new antiviral drug are ongoing [18], no effective antiviral drug against JE is available at present.

JEV and HCV belong to the family *Flaviviridae*, and NTZ inhibits the replication of HCV both in cell culture [14] and in patients [12]. The emergence of NTZ with broad-spectrum antiviral properties prompted us to evaluate its antiviral activity against JEV. We found that NTZ inhibited the replication of JEV in cultured cells and reduced mortality in mice challenged with a lethal dose of JEV.

## Results

### NTZ inhibits the JEV-induced cytopathic effect (CPE)

**Figure 2:**
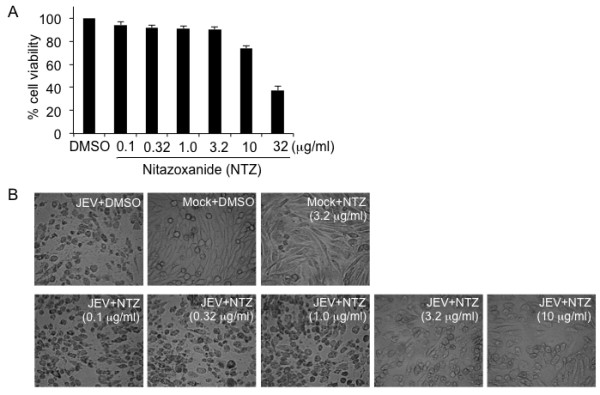
**Analysis of the effect of NTZ on the formation of JEV-induced CPE. (A)** BHK-21 cells were treated with the indicated concentrations of NTZ for 48 h and the cytotoxicity of NTZ was analyzed by MTT assay. DMSO-treated cells were used as a control. The data are means with SD from three independent experiments. **(B)** BHK-21 cells were infected with JEV at a MOI of 0.001. After a 1 h adsorption period, the cells were treated with NTZ at the indicated concentrations and incubated for 48 h. The JEV-induced CPE was examined under a light microscope.

To measure the cytotoxicity of NTZ to baby hamster kidney (BHK-21) cells that are susceptible to JEV infection, BHK-21 cells were treated with NTZ at various concentrations ranging from 0.1 to 32 μg/ml and incubated for 48 h. The cell viability was analyzed by a 3-(4,5-dimethylthiazol-2-yl)-2,5-diphenyltetrazolium bromide (MTT) assay. The 50% cytotoxic concentration (CC_50_) that reduced the proliferation of exponentially growing BHK-21 cells by 50% was 18.59 ± 0.31 μg/ml (Figure 2A). It is known that replication of JEV results in the formation of a CPE in BHK-21 cells. To determine whether NTZ interferes with the formation of JEV-induced CPE, NTZ at concentrations from 0.1 to 10 μg/ml was added to BHK-21 cells after a 1 h JEV adsorption period and the cells were incubated for 48 h. The effect of NTZ on the inhibition of JEV-induced CPE formation was visualized under a light microscope. As shown in Figure 2B, a severe CPE was visible in JEV-infected cells that were treated with dimethyl sulfoxide (DMSO) (JEV + DMSO panel), while in the JEV-infected cells that were treated with NTZ (JEV + NTZ panels), the formation of CPE was reduced by treatment with NTZ in a dose-dependent manner. No CPE was observed in the mock-infected cells that were treated with NTZ (Mock + NTZ panel) or DMSO (Mock + DMSO panel). Although treatment of the mock-infected cells with NTZ or DMSO led to a slight morphological change in cells, these changes were quite different from the JEV-induced CPE. These data implied that NTZ is able to inhibit the replication of JEV in BHK-21 cells.

### NTZ inhibits the replication of JEV in BHK-21 cells

**Figure 3:**
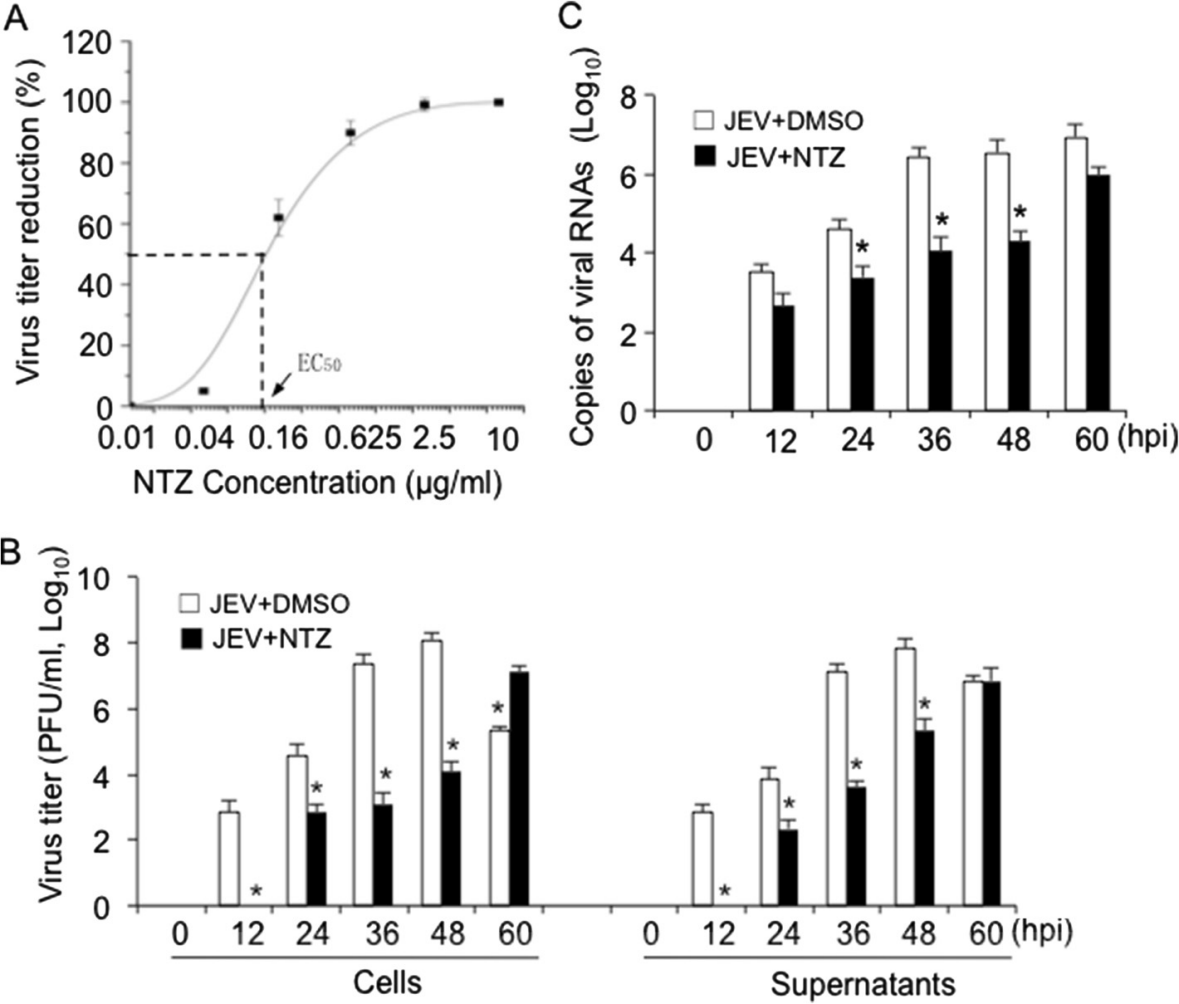
**Analysis of the effect of NTZ on JEV replication in BHK-21 cells.** BHK-21 cells were infected with JEV at a MOI of 0.001 and treated with NTZ at the indicated concentrations. **(A)** The cells were incubated for 48 h and harvested for virus titration. The reduction in virus titer was calculated and plotted. The *x*-axis is in a base 2 logarithmic scale. **(B)** The JEV-infected cells were treated with NTZ at 3 μg/ml and incubated for the indicated times. The virus titers in the cells and supernatants were determined by a plaque assay, respectively. **(C)** The JEV-infected cells were treated with NTZ at 3 μg/ml and incubated for the indicated times. The number of RNA copies of viral C gene in the cells was determined by qRT-PCR. The data are means with SD from three independent experiments. *, *p* < 0.05 compared with the DMSO-treated cells. hpi, hours post-infection.

To determine whether NTZ inhibits the replication of JEV, the JEV-infected BHK-21 cells were treated with NTZ at various concentrations ranging from 0.01 to 10 μg/ml for 48 h. The JEV-infected cells that were parallelly treated with DMSO were used as a control. The titers of virus present in the cell lysates and supernatants were determined by a plaque assay and the reduction in the virus titer was calculated. An inhibition curve of JEV in the presence of a serial dilutions of NTZ is shown in Figure 3A. The 50% effective concentration (EC_50_) of NTZ was 0.12 ± 0.04 μg/ml. The chemotherapeutic index (CTI) calculated as a ratio СC_50_/EC_50_ was 154.92.

To further characterize the antiviral property of NTZ, the JEV-infected BHK-21 cells were treated with NTZ at a concentration of 3 μg/ml and incubated for the indicated times (Figure 3B). The NTZ concentration of 3 μg/ml was chosen because at this concentration NTZ cytotoxicity had no significance and approximately 100% of virus titer reduction was observed (Figure 2A and Figure 3A). The JEV-infected cells that were parallelly treated with DMSO were used as a control. The titers of virus in the cells and in the supernatants were measured by a plaque assay, respectively. In the DMSO-treated cells, the infectious progeny viruses, both in the cells and the supernatants, were detectable from 12 h post-infection, while they were not detectable until 24 h post-infection in the NTZ-treated cells. The viral yields from the NTZ-treated cells were significantly reduced at 12, 24, 36 and 48 h post-infection compared with the DMSO-treated cells (Figure 3B). A decline in viral titer was observed 60 h post-infection in the DMSO-treated cells. This decrease was probably due to serious necrocytosis/apoptosis of the cells induced by rapid viral replication, which contributes to the decline in virus infectivity in addition to temperature that influences the infectivity and stability of extracellular viruses. The number of intracellular RNA copies of the viral C gene in the NTZ-treated and DMSO-treated cells was determined by a quantitative real-time reverse transcription-polymerase chain reaction (qRT-PCR). As shown in Figure 3C, treatment of JEV-infected cells with 3 μg/ml NTZ significantly reduced the number of copies of viral RNA compared with the DMSO-treated cells.

**Figure 4:**
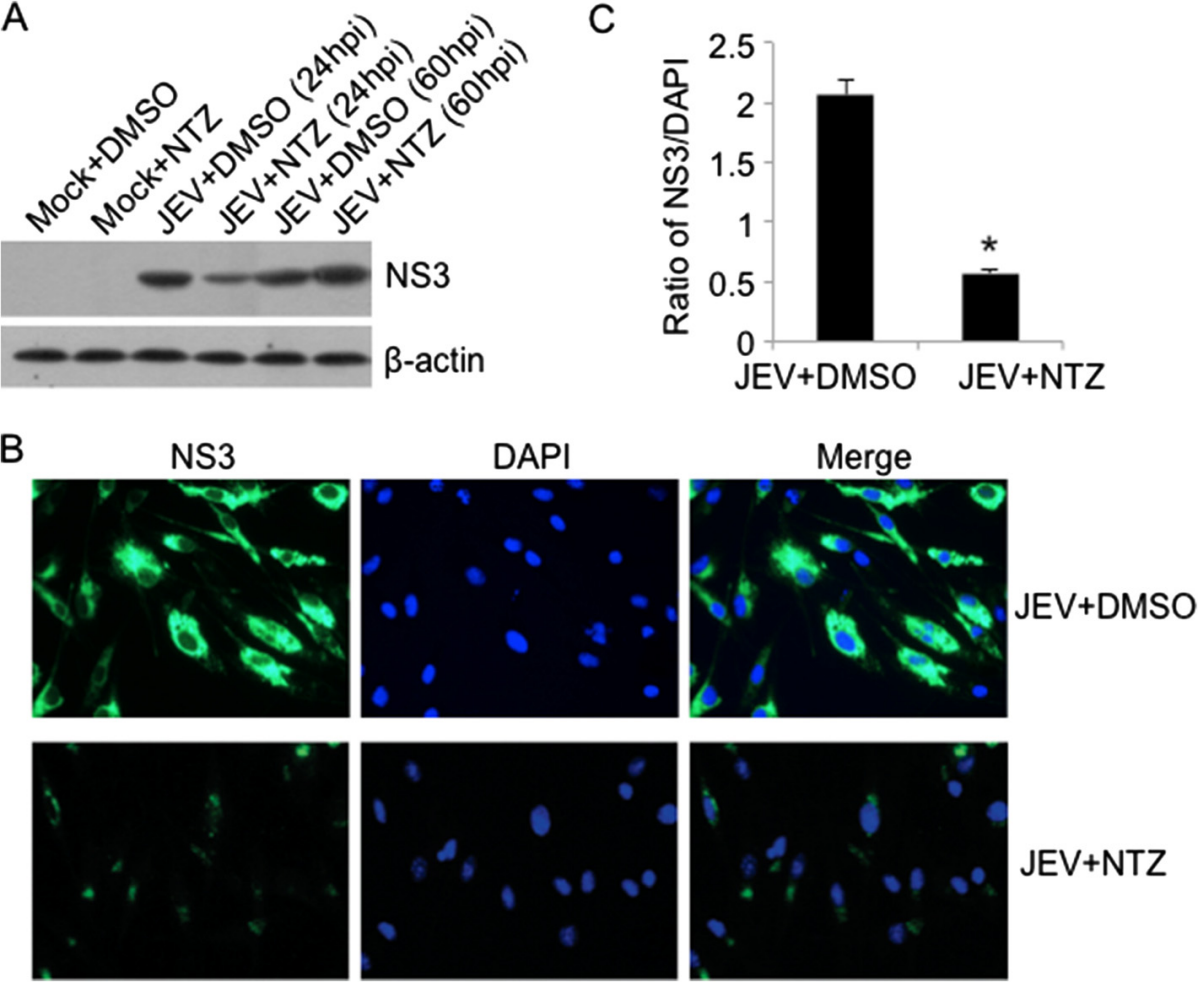
**Detection of viral NS3 protein in NTZ-treated cells.** BHK-21 cells were infected with JEV at a MOI of 0.001 and treated with NTZ at 3 μg/ml. The cells were harvested at 24 or 60 h post-infection (hpi). **(A)** The same amount (20 μg) of cell lysate was loaded in each lane. The expression of viral NS3 protein was examined by western blot. **(B)** The viral NS3 protein (green fluorescence) was detected at 24 hpi by immunofluorescence analysis. The cells were also stained for DNA with 4′, 6′-diamidino-2-phenylindole (DAPI, blue fluorescence). The Merge panels show the superimposed images. **(C)** Fluorescence intensity of NS3 and DAPI was quantified using the software ImageJ. The ratio of fluorescence intensity between NS3 and DAPI was calculated and plotted. The data are means with standard errors from three independent experiments. *, *p* < 0.01 between the groups tested.

The inhibitory effect of NTZ on the replication of JEV was further confirmed at viral protein level. The expression of viral NS3 protein in the NTZ-treated and DMSO-treated cells was detected by western blot and immunofluorescence analysis. As shown in Figure 4A, the abundance of viral NS3 protein in the NTZ-treated cells (JEV + NTZ) at 24 h post-infection was remarkably less than that in the DMSO-treated cells (JEV + DMSO). In the immunofluorescence analysis, although the numbers of NS3-positive cells (green fluorescence) among the NTZ-treated cells (JEV + NTZ panels) were similar to those detected among the DMSO-treated cells (JEV + DMSO panels), the strength of the fluorescence signals in the NTZ-treated cells was remarkably lower than that in the DMSO-treated cells (Figure 4B and 4C). Taken together, these data suggested that NTZ inhibited the replication of JEV.

### NTZ inhibits JEV replication at the early-mid stage of viral infection

**Figure 5:**
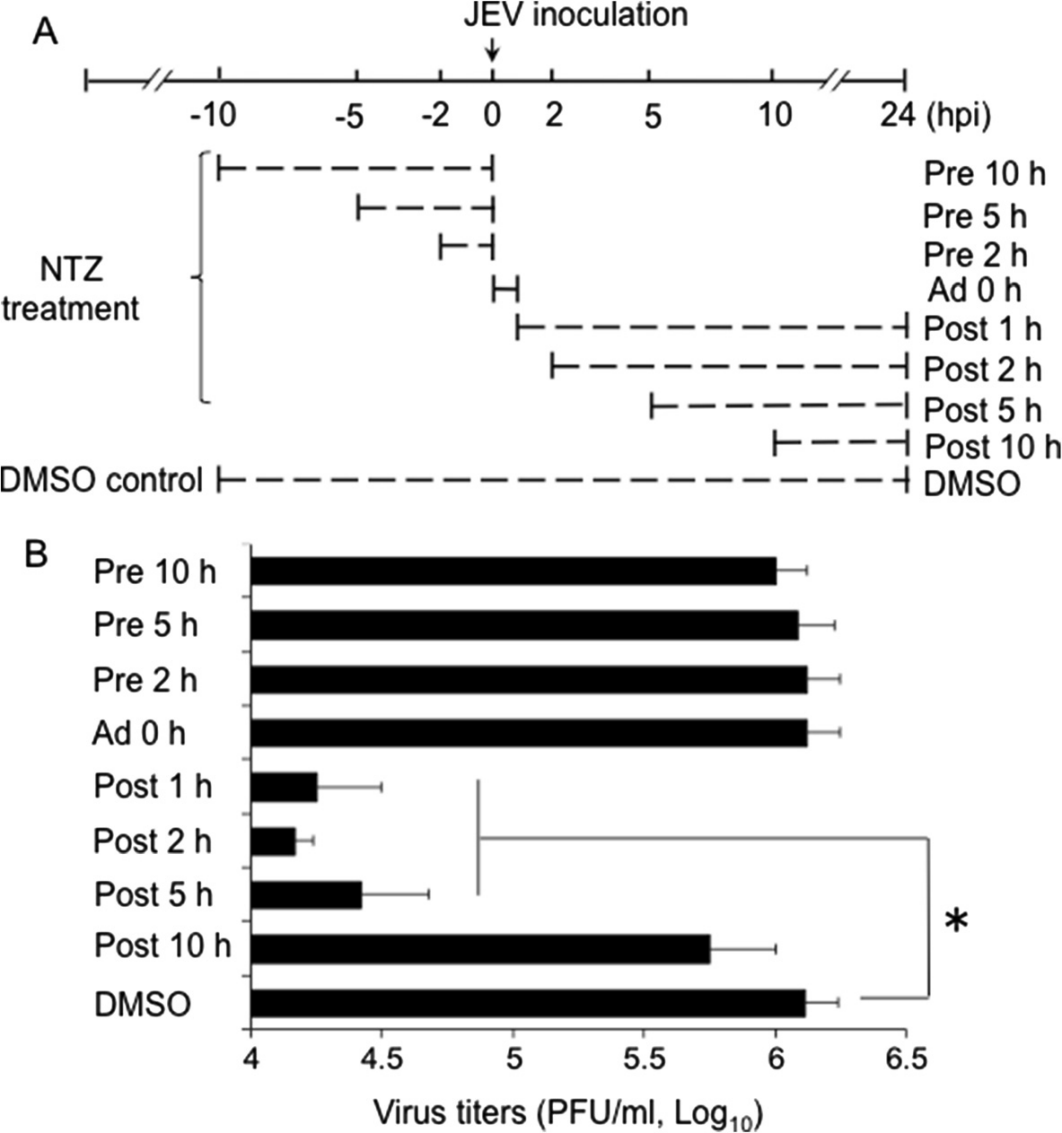
**Inhibition of JEV replication *in vitro *by NTZ at the early-mid stage of viral infection. (A)** Schematic representation of the experimental design. BHK-21 cells were treated with 3 μg/ml NTZ at the indicated times before infection (Pre 10 h, Pre 5 h and Pre 2 h), during the adsorption period (Ad 0 h), and after the adsorption period (Post 1 h, Post 2 h, Post 5 h and Post 10 h). **(B)** The virus titer in each group was determined 24 h post-infection by a plaque assay and plotted. The data are means with SD from three independent experiments. *, *p* < 0.05 between the groups tested.

To determine the stage at which NTZ affected JEV replication, NTZ at a concentration of 3 μg/ml was added into BHK-21 cells at different time points before viral infection (Pre 10 h, Pre 5 h and Pre 2 h), during viral adsorption (Ad 0 h), at the early stage of viral infection (Post 1 h and 2 h), at the mid stage of viral infection (Post 5 h) and at the late stage of viral infection (Post 10 h) (Figure 5A). The viral titers in each group were assayed 24 h post-infection. As shown in Figure 5B, pretreatment of cells with NTZ up to 10 h before viral infection had no effect on JEV replication (Pre 2 h, 5 h and 10 h). Moreover, NTZ treatment only during the adsorption period did not inhibit virus replication (Figure 5B, Ad 0 h), indicating that NTZ was not directly affecting viral infectivity, adsorption and entry into target cells. In contrast, NTZ treatment initiated at the early stage of viral infection (Post 2 h) was the most effective in inhibiting virus replication (Figure 5B, Post 2 h). NTZ treatment started at the mid stage of viral infection (Post 5 h) was relatively less effective, but still able to significantly inhibit virus replication (Figure 5B, Post 5 h), whereas NTZ was ineffective when administered at the late stage of viral infection (Figure 5B, Post 10 h). Taken together, these data suggested that NTZ inhibited JEV replication *in vitro* at the early-mid stage of viral infection.

### NTZ reduces the mortality of mice challenged with a lethal dose of JEV

**Figure 6:**
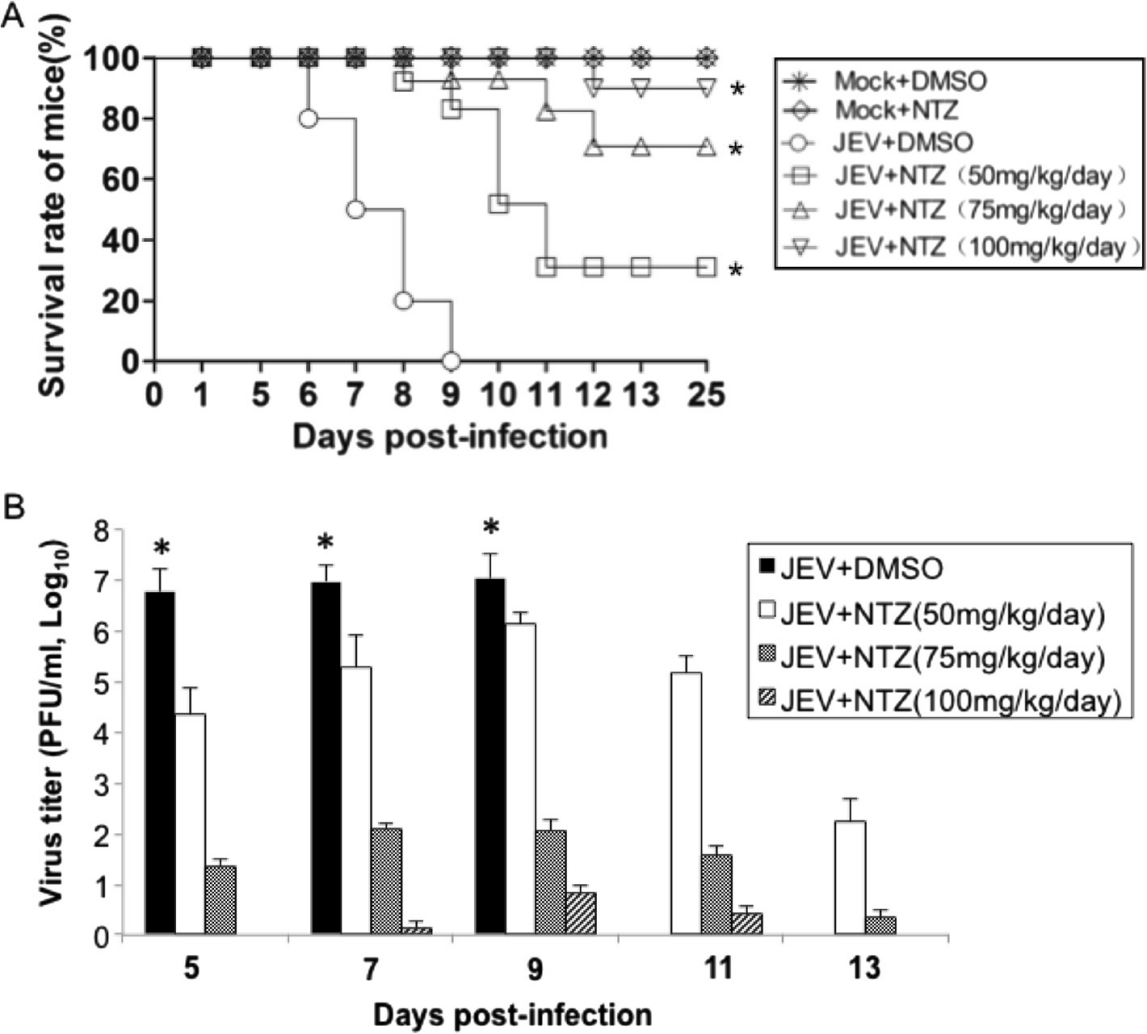
**Analysis of the protective effect of NTZ on mice challenged with a lethal dose of JEV.** Mice (10 mice/group) were infected intraperitoneally with 6×10^4^ PFU of JEV and treated with NTZ from 1 day post-infection. NTZ was administered daily at the indicated doses (50, 75 or 100 mg/kg/day) by intragastric administration for up to 25 days. **(A)** The mice were monitored daily for morbidity and mortality. The daily survival rates were plotted. *, *p* < 0.0001 between the group JEV + DMSO and group JEV + NTZ tested by the Gehan-Breslow-Wilcoxon test. **(B)** The brain tissues were collected from the mice euthanized on the indicated days after JEV infection. The virus titer in each group was determined by a plaque assay and plotted. The data are means with SD from three independent experiments. *, *p* < 0.05 compared with the NTZ-treated groups.

To evaluate the protective effect of NTZ on mice challenged with a lethal dose of JEV, NTZ was administered intragastrically at the indicated doses from 1 day post-infection, daily, for up to 25 days. The mice that were infected with JEV and received a placebo (DMSO) treatment (group JEV + DMSO) started to show the clinical signs of JE including limb paralysis, restriction of movements, piloerection, body stiffening and whole body tremors, from 5 days post-infection, and all mice (10/10 mice) died within 9 days post-infection. In contrast, the mice that were infected with JEV and received NTZ treatment (group JEV + NTZ, 100 mg/kg/day) showed the clinical signs of JE from 11 days post-infection, among these 10 mice, 1 died within 12 days post-infection and 9 survived the experimental period (25 days) (Figure 6A). The NTZ-mediated protection appeared to be dose dependent, as the infected mice receiving 50 mg/kg/day, 75 mg/kg/day and 100 mg/kg/day NTZ led to 30%, 70% and 90% mice survival, respectively (Figure 6A). These data suggested that NTZ treatment reduced the mortality of JEV-infected mice and protected mice from a lethal dose challenge of JEV. The mice that were mock-infected with JEV and received NTZ treatment (group Mock + NTZ) showed no detectable signs of abnormal behavior, similarly to the mice that were mock-infected and received DMSO treatment (group Mock + DMSO) (Figure 6A). Analysis of JEV titers in the brain samples from the experimental mice indicated that NTZ treatment significantly reduced the virus load in the brain from the group JEV + NTZ compared with that from group JEV + DMSO (Figure 6B). The brain samples from the experimental mice were examined for the presence of viral NS3 protein by immunohistochemistry. The viral NS3 protein was stained as brown deposits in the cytoplasm of neuronal cells (Additional file 1, arrowed cells). The brain sections from the JEV + DMSO group of mice showed remarkably higher numbers of NS3-stained positive cells than the sections from the JEV + NTZ group. No NS3-stained positive cells were detected in the sections from the Mock + NTZ or Mock + DMSO groups of mice.

## Discussion

NTZ is an anti-infective licensed in the United States for the treatment of parasitic gastroenteritis [1-3]. This drug has been demonstrated to be safe and effective even when administered over a year [8]. NTZ has been considered to be a new class of broad-spectrum antiviral drugs [11]. Given the fact that there is an urgent need for an effective drug against JEV and that there is currently no effective antiviral agent available for the treatment of JE, we tested the antiviral effect of NTZ on JEV infection in cell and mouse models. We found that NTZ was able to inhibit the replication of JEV in BHK-21 cells in a dose-dependent manner (Figure 2 and Figure 3). NTZ also reduced the mortality of mice challenged with a lethal dose of JEV (Figure 6).

We observed that NTZ significantly inhibited the replication of JEV in BHK-21 cells in a dose dependent manner with EC_50_ value of 0.12 ± 0.04 μg/ml (Figure 3A), whereas the EC_50_ for influenza A virus (PR8 strain) is 1.0 μg/ml [9]. As a new class of broad-spectrum antiviral drugs, NTZ has been speculated to target host functions that are essential for viral replication [9,14]. In the case of HCV infection, NTZ increases eIF2α phosphorylation, a modification known to mediate host antiviral defenses [14]. However, in the case of influenza virus infection, NTZ prevents the maturation of viral HA possibly by blocking HA trafficking between the endoplasmic reticulum and the Golgi complex [9]. In our study, we found that NTZ exerted its antiviral activity *in vitro* at the early-mid stage of viral infection (Figure 5), indicating that NTZ does not affect JEV infectivity, adsorption or entry into target cells. However, the exact mechanism of how NTZ inhibited JEV replication needs to be further explored.

Although there are currently no effective drugs available for the treatment of JE, attempts to develop new antiviral drugs are ongoing. For example, peptide-conjugated phosphorodiamidate morpholino oligomers (PPMOs) have recently been reported which are antisense agents targeting the 3′ cyclization sequence of JEV, they have been shown to significantly inhibit the replication of JEV in cells and in a mouse model [19]. In comparison with previously proposed therapeutic agents, such as PPMOs, siRNA [20,21], arctigenin [22] and isatis indigotica extracts [23], NTZ is a licensed, safe drug and is therefore a promising candidate for use as an anti-JEV therapeutic agent. Further studies into the mechanism of action and the efficacy of NTZ are currently underway in our laboratory.

## Conclusions

The anti-JEV activity of NTZ was evaluated in cultured cells and in a mouse model. NTZ significantly inhibited the replication of JEV in BHK-21 cells in a dose dependent manner with EC_50_ value of 0.12 ± 0.04 μg/ml. NTZ was found to exert its anti-JEV effect *in vitro* at the early-mid stage of viral infection. The anti-JEV effect of NTZ was also demonstrated *in vivo*, where 90% of mice that were treated with NTZ were protected from a lethal challenge dose of JEV. Both our *in vitro* and *in vivo* data indicated that NTZ has anti-JEV activity, suggesting the potential application of NTZ in the treatment of JE.

## Materials and methods

### Virus, cells and NTZ administration

JEV strain (SH-JEV01) [24] was grown in 3-day-old BALB/c mice and titrated by a plaque assay using BHK-21 cells as described below. BHK-21 (C-13, ATCC number CCL-10) cells were maintained in Dulbecco’s modified Eagle’s medium (DMEM) supplemented with 10% fetal bovine serum (FBS) at 37°C in an atmosphere containing 5% CO_2._

NTZ (purity ≥ 98%) was purchased from Sigma–Aldrich (Sigma, St. Louis, MO, USA) and was dissolved in culture-grade DMSO (Sigma) at a concentration of 50 μg/μl. NTZ solution was added immediately after a 1 h adsorption period and was kept in the culture medium for the duration of the experiment, unless specified otherwise. Controls received equal amounts of DMSO (final concentration ≤ 0.06%), which did not affect cell viability or virus replication.

### Cytotoxicity test

BHK-21 cells were seeded in a 96-well plate at a density of 5 × 10^3^ cells per well. Following 24 h of incubation, the cells were treated with NTZ at various concentrations ranging from 0.1 to 32 μg/ml at 37°C for 48 h. Cells treated with DMSO alone were used as a control. The cellular toxicity of NTZ was assessed using MTT assay (Roche, Mannheim, Germany). Cell viability was calculated as a percentage of the total number of viable DMSO-treated control cells. The CC_50_, which is defined as the concentration that inhibits the proliferation of exponentially growing cells by 50%, was calculated as described [25].

### Viral infection and titration

BHK-21 cells were seeded at 1 × 10^6^ cells per well on six-well plates. Cultures were inoculated with JEV at a multiplicity of infection (MOI) of 0.001 when the cells reached approximately 80–90% confluence. The inoculum was removed following a 1 h incubation period, and cells were washed twice with phosphate-buffered saline (PBS). Cultures were further incubated in DMEM with 2% FBS for the indicated times. Following incubation, the cells and the culture supernatants were collected and stored at -70 °C until further use.

The plaque assay for JEV infectivity was performed as described [26]. Briefly, BHK-21 cells were seeded in six-well plates and incubated overnight. The medium was replaced with PBS containing serially diluted JEV. The cells were incubated at 37°C for 4 h. After the adsorption period, the inoculum was replaced with DMEM containing 2% FBS and 1% low-melting-point agarose and incubated for 5 days. On 5 days post-inoculation, the cells were fixed with 1% formaldehyde overnight to penetrate the agarose. The cells were stained with 0.05% neutral red for 1 h at room temperature after removing the overlaid agarose. The plaques were then counted, and infectivity was expressed as plaque forming units (PFU)/ml.

### qRT-PCR

Total RNA was isolated from cells using the QIAamp viral RNA minikit (Qiagen, Hilden, Germany), according to the manufacturer’s protocol. One microgram of total RNAs isolated from JEV-infected cells was used for synthesis of cDNA using AMV reverse transcriptase (TaKaRa, Otsu, Japan). The qRT-PCR for analysis of JEV replication was performed using Premix Ex Taq™ (TaKaRa), according to the manufacturer’s protocol. Briefly, reactions were prepared in 20 μl containing 1 μl of cDNA, 10 μl of SYBR Premix Ex Taq™ (2×), and 0.2 μM of specific primers. The amplification parameters were: 2 min at 95°C, followed by 40 cycles of 15 sec at 95°C, and 60 sec at 60°C. The sequences of the primers used to amplify 181 bp of the gene encoding core protein C (C) of JEV were: 5′-AAAAACCAGGAGGGCCCGG-3′(nucleotide position 103–121) and 5′-TTGGTCGGGGCTAATGCTGTAAA-3′(nucleotide position 283–261). These primers were designed according to the sequence of JEV strain SA14 (GenBank accession No. U14163). Quantification was achieved by relating the cycle threshold (Ct) value of virus to the Ct value on a standard curve of a measured number of copies of the JEV C gene cloned into pMD18-T vector which the resulting plasmid was named pM-C. The quantitative standard curve for the determination of JEV RNAs copy number was created by quantitative real-time PCR of standard plasmid pM-C preparations at serial dilutions of 10^6^, 10^5^, 10^4^, 10^3^, 10^2^ and 10^1^ copies/μl. The number of JEV RNAs copies was calculated by Eppendorf Mastercycler ep realplex 2.2 software automaticlly.

### Analysis of the antiviral effect of NTZ in BHK-21 cells

BHK-21 cells in six-well plates were infected with JEV at a MOI of 0.001. After a 1 h adsorption period, the cells were treated with NTZ at concentrations ranging from 0.01 to 10 μg/ml and incubated at 37°C for 48 h or the indicated times. The virus yield was determined by a plaque assay and qRT-PCR. The reduction in the virus titer was calculated as follows:% virus titer reduction = [1-(PFU_JEV+NTZ_/PFU_JEV+DMSO_)] × 100. The EC_50_ that is defined as the concentration offering 50% inhibition of viral yield in cells was calculated as described [25].

### Western blot, immunofluorescence and immunohistochemistry analysis

The expression of viral NS3 in JEV-infected cells was examined by western blot and immunofluorescence analysis as described [24,27]. The fluorescence intensity of cells was quantified using the software ImageJ that is a Java-based image processing program developed at the National Institutes of Health (http://rsbweb.nih.gov/ij/index.html). The immunohistochemistry for analysis of viral NS3 in brain of JEV-infected mice was performed as described [24].

### Analysis of the antiviral effect of NTZ in a mouse model

Three-week old female Chinese Kunming mice (12–14 g body weight) were purchased from Shanghai SLAC Laboratory Animal Co., Ltd (Shanghai, China) and were randomly divided into six groups (10 mice/group). Group JEV + NTZ was infected with JEV and received NTZ treatment (50, 75 or 100 mg/kg/day). Group JEV + DMSO was infected with JEV and received a placebo (DMSO) treatment. Group Mock + NTZ was mock-infected with JEV and received NTZ treatment. Group Mock + DMSO was mock-infected with JEV and received a placebo (DMSO) treatment. For infection, mice were infected intraperitoneally with 6×10^4^ PFU of JEV (containing 50 × LD_50_ of JEV). For NTZ treatment, NTZ was dissolved in DMSO and administered intragastrically by gavage, in which a feeding needle was introduced into the esophagus and NTZ was delivered directly into the stomach. NTZ was tested at a total dose of 50, 75 or 100 mg/kg/day, and was consecutively administered from 1 day post-infection, daily, for up to 25 days. The mice were monitored daily for morbidity and mortality. The mice that showed neurological signs were euthanized according to the Guidelines on the Humane Treatment of Laboratory Animals (Ministry of Science and Technology of the People’s Republic of China, Policy No. 2006 398). The animal experiments were in compliance with the Guidelines on the Humane Treatment of Laboratory Animals (Policy No. 2006 398) and were approved by the Institutional Animal Care and Use Committee at the Shanghai Veterinary Research Institute, Chinese Academy of Agricultural Science.

### Statistical analysis

All experiments were carried out in triplicate. The measured values are expressed as the means with standard deviations (SD). Significance was analyzed using the Student’s *t*-test or the Gehan-Breslow-Wilcoxon test. Value of *p* < 0.05 was considered significant.

## Abbreviations

NTZ: Nitazoxanide; EC50: 50% effective concentration; CC50: 50% cytotoxic concentration; CTI: Chemotherapeutic index; MTT: 3-(4,5- dimethylthiazol-2-yl)-2,5-diphenyltetrazolium bromide; MOI: Multiplicity of infection; PFU: Plaque forming units.

## Competing interest

The authors declare that they have no competing interests.

## Authors’ contributions

ZXS and JCW carried out most of the experiments and wrote the manuscript. XFD and SQL grown and titrated JEV virus. YFQ and DHS performed the qRT-PCR and MTT assay. BBL helped in the mouse infection. KYZ and FQX helped in NTZ preparation and experimental design. XDW helped in western blot and immunofluorescence analysis. ZYM designed the experiments and revised the manuscript. All of the authors read and approved the final version of this manuscript.

## Supplementary Material

Additional file 1 **The brain tissues were collected from the mice euthanized on 9 days after JEV infection and were stained immunohistochemically for viral NS3 protein, which stains as brown deposits in the cytoplasm of neuronal cells (arrowed cells).** JEV+NTZ, mice were infected with JEV and received NTZ (100 mg/kg/day) treatment. JEV+DMSO, mice were infected with JEV and received a placebo (DMSO) treatment. Mock+NTZ, mice were mock-infected with JEV and received NTZ (100 mg/kg/day) treatment. Mock+DMSO, mice were mock-infected with JEV and received DMSO treatment. Bar, 100 μm. Click here for file
