# Supplementary figures and images for: Nitazoxanide inhibits the replication of Japanese encephalitis virus in cultured cells and in a mouse model
Source: Virol J. 2014 Jan 23;11:10. doi: 10.1186/1743-422X-11-10 (PMC3927656; doi:10.1186/1743-422X-11-10)

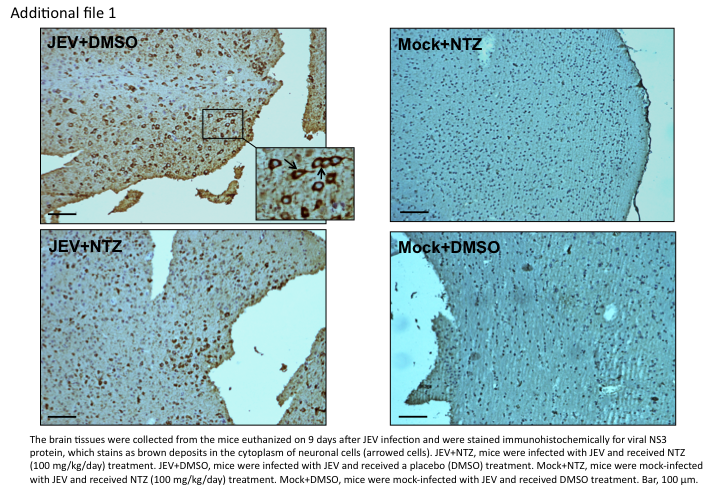

Supplement: Additional file 1 — The brain tissues were collected from the mice euthanized on 9 days after JEV infection and were stained immunohistochemically for viral NS3 protein, which stains as brown deposits in the cytoplasm of neuronal cells (arrowed cells). JEV+NTZ, mice were infected with JEV and received NTZ (100 mg/kg/day) treatment. JEV+DMSO, mice were infected with JEV and received a placebo (DMSO) treatment. Mock+NTZ, mice were mock-infected with JEV and received NTZ (100 mg/kg/day) treatment. Mock+DMSO, mice were mock-infected with JEV and received DMSO treatment. Bar, 100 μm. [file 1743-422X-11-10-S1.tiff]
